# Improving Bovine Brucellosis Diagnostics: Rapid, Accurate Detection via Blood Serum Infrared Spectroscopy and Machine Learning

**DOI:** 10.1021/acsomega.5c00504

**Published:** 2025-05-24

**Authors:** Thiago Franca, Miller Lacerda, Camila Calvani, Kelvy Arruda, Ana Maranni, Gustavo Nicolodelli, Sivakumaran Karthikeyan, Bruno Marangoni, Carlos Nascimento, Cicero Cena

**Affiliations:** 1 Optics and Photonic Lab (SISFOTON-UFMS), 54534 UFMS−Universidade Federal de Mato Grosso do Sul, Av. Costa e Silva s/n, Campo Grande, MS 79070-900, Brazil; 2 28117 UFSC−Universidade Federal de Santa Catarina, Rua Eng Argonomico Andrei Cristian s/n, Florinópolis, SC 88040-900, Brazil; 3 Department of Physics, Dr. Ambedkar Government Arts College, Chennai, Tamilnadu 600039, India

## Abstract

Diagnosing bovine brucellosis is a major challenge due to its significant economic impact, causing losses in meat and dairy production and its potential to transmit to humans. In Brazil, disease control relies on diagnosis, animal culling, and vaccination. However, existing diagnostic tests, despite their quality, are time-consuming and prone to false positives and negatives, complicating effective control. There is a critical need for a low-cost, fast, and accurate diagnostic test for large-scale use. Spectroscopy techniques combined with machine learning show great promise for improving diagnostic tests. Here, we explore the potential use of FTIR (Fourier transform infrared) spectroscopy and machine learning algorithms to provide a rapid, accurate, and cost-effective diagnostic method for Brucella abortus. This study explored the use of FTIR spectroscopy on bovine blood serum in liquid and dried forms to develop a new photodiagnosis method. Eighty bovine blood serum samples (40 infected and 40 control animals) were analyzed. Initially, the FTIR data were pretreated using the standard normal deviate method to remove baseline deviations. Principal component analysis was then applied to observe clustering tendencies, and the further selection of principal components improved clustering. Using support vector machine algorithms, the predictive models achieved overall accuracies of 95.8% for dried samples and 91.7% for liquid samples. This new methodology delivers results in about 5 min, compared to the 48 h required for standard diagnostic methods. These findings demonstrate the viability of this approach for diagnosing bovine brucellosis, potentially enhancing disease control programs in Brazil and beyond.

## Graphical abstract

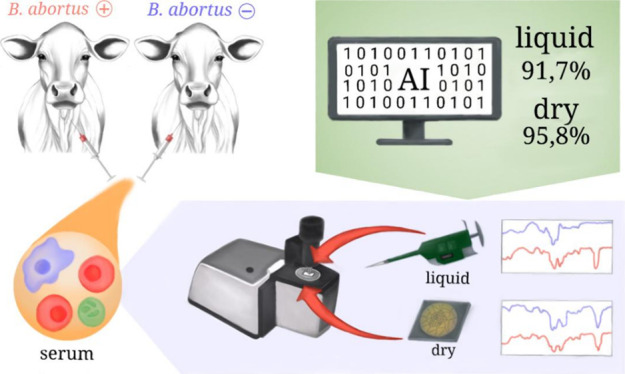

## Introduction

Bovine brucellosis is a highly infectious zoonotic disease caused primarily by the bacterium Brucella abortus, which affects cattle and can also impact other livestock and humans.^1^ The disease causes reproductive failures in cattle, such as abortions, stillbirths, and infertility, leading to significant economic losses in the livestock industry.^1^ The disease management and control strategies depend on the rapid and accurate identification of infected animals.^1,2^ Despite advances in bovine brucellosis diagnostic methods, we are still seeking an easily implemented diagnostic method, portable for field applications, that may offer faster and more reliable results.^2,3^

Brucellosis triggers a complex immune response in cattle, characterized by robust activation of both innate and adaptive immunity. Briefly, the innate immune response is the body’s first line of defense against infections and injuries. It provides a rapid, nonspecific response using physical and chemical barriers, immune cells (such as macrophages, neutrophils, and natural killer cells), and proteins. This response also involves inflammation to isolate and combat pathogens and uses pattern recognition receptors to detect common features of pathogens. It is essential for immediate defense and activation of the more specific adaptive immune response. The adaptive immune response is a targeted defense mechanism that develops following exposure to a pathogen. It involves the activation of lymphocytes, specifically B cells (bone marrow-derived cells) and T cells (thymus-derived cells), which recognize and respond to specific antigens. B cells produce antibodies that neutralize pathogens, while T cells destroy infected cells or help other immune cells.^4–6^

As a result, this immune response leads to elevated levels of pro-inflammatory cytokines—signaling proteins such as tumor necrosis factor-alpha (TNF-α), interleukin-1 (IL-1), and interleukin-6 (IL-6), which play key roles in promoting inflammation and coordinating the body’s defense mechanisms.^7^ The serum of infected cattle typically shows elevated levels of specific antibodies against *Brucella* antigens. The detection of these antibodies is crucial for diagnosing the disease, although cross-reactivity with other infections can complicate interpretations.^2^ The number of acute phase proteins (APPs) such as haptoglobin and serum amyloid A gets higher. These proteins serve as biomarkers for inflammation and can help assess the severity of the infection.^8^ The immune response may also cause alterations in serum electrolytes and total protein levels, reflecting the systemic effects of the infection and the body’s response to it.^9^

This scenario is fruitful for using molecular optical spectroscopy to investigate biofluid properties, whose characteristics can lead to a data analysis protocol to find a new photodiagnostic method. Molecular optical spectroscopies, such as Fourier transform infrared (FTIR) and Raman spectroscopy, are very sensitive to small changes in the sample composition and have been used for the development of many photodiagnosis methods.^10–13^ The main advantage of FTIR or Raman spectroscopy lies in the robustness of the technique, rapid response (few seconds), and easy implementation, without the need for extensive preparation, making them less labor-intensive and low cost. At the same time, the importance of developing new methods for diagnosing brucellosis lies in the disease’s impact on public health, agriculture, and economic stability.

A previous study investigated serum samples from Brucella-infected sheep by Fourier transform infrared (FTIR) spectroscopy combined with principal component analysis–linear discriminant analysis (PCA-LDA). According to the study, the spectral ranges of 3700–3090 and 3000–2800 cm^–1^, usually assigned to lipid molecules, achieved 100% sensitivity, specificity, and accuracy.^14^ The FTIR-PCA-LDA method outperformed traditional diagnostic methods, such as indirect enzyme-linked immunosorbent assay (ELISA) and serum fluorescence polarization assay (FPA), in terms of sensitivity, specificity, and accuracy. These findings suggest that the FTIR-PCA-LDA method provides high diagnostic accuracy for distinguishing Brucella-infected sheep from healthy sheep. The classification models were validated by using independent sample sets. The performance of the models was described by using receiver operating characteristic (ROC) and area under the curve (AUC) parameters.^14^

A prior study on bovine brucellosis successfully applied ultraviolet-visible spectroscopy (UV–vis) combined with machine learning (ML) algorithms for disease detection, demonstrating the potential of spectroscopic techniques in diagnostics. Due to the nature of UV–vis spectroscopy, to enhance the detection of sample differentiation, the antigen for B. abortus infection was diluted in saline containing phenol. Then, 2 μL of the serum–antigen mixture was characterized in the 200–300 nm range after 3 min of incubation at room temperature. After data preprocessing, the results obtained from principal component analysis (PCA) did not suggest any tendency of group separation; to improve such characteristics, the authors applied a feature selection recursive feature elimination (RFE) algorithm to select the principal components (PCs) that mostly contributed to group separation. After the selection of three PCs (PC3, PC4, and PC14) that contributed mostly to group classification, the best prediction model was built by using the *k*-nearest neighbor (KNN) algorithm with the cosine function, achieving an overall accuracy of 95.9% in the leave-one-out cross-validation (LOOCV) test. The analytical sensitivity was 92%, and the specificity was 100%. In external validation, the model showed an accuracy of 84.4%, sensitivity of 75%, and specificity of 94%. The proposed UV–vis/ML method effectively distinguished between positive and negative samples for antibodies against B. abortus in cattle, reducing the diagnostic time to about 5 min, compared to the 48 h required by traditional methods such as the buffered acidified antigen test (BAAT), the 2-mercaptoethanol test (2-ME), and the standard agglutination test (SAT).^15^

Despite the great potential demonstrated by optical spectroscopy combined with machine learning algorithms for disease diagnosis, the method also faces limitations. If the disease does not cause significant alterations in the sample to be analyzed, then usual strategies involving PCA analysis and regular machine learning algorithms, such as KNN, SVM (support vector machine), and LDA (linear discriminant analysis), may not achieve high accuracy. For instance, in an animal model study, to diagnose cutaneous leishmaniasis in female BALB/c mice, blood serum analysis by FTIR spectroscopy, followed by PCA analysis and the SVM algorithm, only achieved 72% accuracy.^12^

Research has indicated that optical spectroscopy can be used to identify specific biomarkers related to brucellosis such as changes in serum proteins or metabolic profiles. This could lead to the development of point-of-care diagnostic tools that are both effective and easy to use. Combining optical spectroscopy with existing molecular methods, such as polymerase chain reaction (PCR), could enhance diagnostic accuracy and provide a comprehensive approach to brucellosis detection.^16^ In summary, advancing diagnostic methods for brucellosis is crucial for effective disease management and public health safety. Optical spectroscopy offers a novel approach that could improve the speed, sensitivity, and specificity of brucellosis diagnosis, addressing the current limitations in traditional methods. In this study, FTIR data was analyzed using machine learning algorithms to develop predictive models. By applying these techniques, we achieved over 90% accuracy in sample classification, significantly reducing the subjectivity inherent in current diagnostic tests, which rely on human interpretation.

## 2 Materials and Methods

### 2.1 Sample Description, Preparation, and Data Collection

Eighty bovine blood serum samples were provided by the Federal Agricultural Defense Laboratory-MG (LFDA), which receives samples from all over Brazil for diagnosis under the National Program for the Control and Eradication of Brucellosis and Animal Tuberculosis (PNCEBT).^17^ Therefore, the samples were not originally collected by the LFDA for research purposes. The blood serum originated from cows of various breeds, sexes, weights, and ages. The samples were divided into 40 serum samples from infected animals and 40 from a control group. The infected group was determined as positive for antibodies against B. abortus, according to reference tests recommended by Brazilian legislation (BAAT, 2-ME, and SAT).^16^

Serum was used instead of whole blood to minimize spectral interference from cellular components, particularly red blood cells, which can dominate the FTIR signal and obscure subtle biochemical variations related to the immune response.^10^ The samples were analyzed in both liquid and dried forms in the 1800–900 cm^–1^ range, with a resolution of 4 cm^–1^, and 12 scans, by using a Fourier transform infrared spectroscope, Agilent, Cary 630 model, with an attenuated total reflectance (ATR) accessory.

Dried samples were prepared by casting blood serum onto a silicon oxide (SiO_2_) substrate. A thick sample was obtained after three steps of 20 μL of deposition followed by a drying process at 40 °C for 10 min. The average spectra of the dried samples were collected in three different spots (at the center and two edges of the drop) to mitigate compositional heterogeneity,^18^ and an atmospheric background was obtained before each acquisition. Liquid samples were left to thaw at room temperature, and then, 20 μL was directly deposited on the attenuated total reflectance accessory (ATR) at the FTIR spectrometer; deionized water was used to obtain the background before each spectral acquisition.

### 2.2 Data Pretreatment and Prediction Model Development

The entire data analysis was performed using the Scikit-Learn library (version 1.3.0) in the Python programming language (version 3.11.5).^19^ First, the FTIR raw data was preprocessed using standard normal variate (SNV). SNV is a well-known normalization process that enhances data quality by reducing experimental drift and systematic variations, allowing for more accurate and consistent spectral analysis. Practically, SNV normalizes spectroscopic data by adjusting each spectrum so that its mean is 0 and its standard deviation is 1, facilitating the comparison between different samples and the extraction of relevant features for analytical models.^20^ Due to noise in the spectra of liquid samples, we also applied an IFFT (inverse fast Fourier transform) filter to smoothen the curve.^21^

Second, the FTIR-SNV spectra of each group were divided into 60% for training and 40% for testing and submitted to principal component analysis (PCA).^22^ Here, we apply Hotelling’s T^2^ to remove outliers by measuring the distance of a sample from the multivariate mean, considering the covariance of the variables across the entire sample set.^23^ The PCA is an important analysis to reduce the dimensionality of large data sets, such as spectral data, while preserving as much variability as possible. This is achieved by transforming the original data into a new set of uncorrelated variables, called principal components. As a result, we obtain information regarding the data variance; (i) the score plot provides information about the distribution and clustering of samples, revealing potential separation and grouping patterns of the new data at the principal component space. The separation or grouping depends on the data variance related to each PC; (ii) the contribution of each PC and its meaning can be revealed by analyzing the loading plot, which provides insights into the contribution of each original variable to the principal components’ variance, helping to identify which variables are most influential in the data’s variance and contribute for group separation when it occurs.

Once the potential separation of the groups is explored and revealed by PCA analysis, we can build a prediction model to automatize the sample classification and perform validation tests for the method. In the next step, PCA data (60% samples for each group) were used to train support vector machine (SVM) algorithms, which explore diverse functions (linear, quadratic, and cubic) to construct hyperplanes responsible for separating the groups and then classify new samples according to their classes. The algorithm can optimize the hyperplane characteristics to enhance the accuracy, and hyperparameter *C*, which manages the trade-off between maximizing the margin and minimizing classification errors, was adjusted to balance fitting the training data well while preventing overfitting. Fine-tuning of this hyperparameter was crucial, as their optimal values depend on the data set’s characteristics. The model was regularized using linear and polynomial kernels (degrees 2 and 3) and hyperparameters (*C* = 1, 10, or 100).^24^

The performance of the SVM was evaluated using leave-one-out cross-validation (LOOCV).^25^ In this method, one sample was withdrawn from the data set, and the prediction model was built using the remaining data. The model was then tested on the withdrawn sample. This process was repeated until each sample was removed and tested. The final accuracy was calculated as the average of the accuracy values obtained from each iteration. Finally, a validation test was performed by using the best SVM, function, and hyperparameters found in the LOOCV test by using the remaining 40% of the samples. The results were summarized in a confusion matrix, showing how the samples were classified according to their original label and the overall accuracy of the model.

## 3 Results and Discussion

Figure 1 shows the FTIR-SNV spectra for the bovine blood serum samples. The blue-colored trace represents the control group (noninfected by B. abortus), and the red-colored trace represents the B. abortus-infected group (from here called the brucellosis group). The dried sample (upper spectral set) and the liquid sample (lower spectral set) exhibit significant differences, particularly in the 1650–1500 cm^–1^ range. These differences are primarily due to strong bands associated with C=C and C=O stretching, as well as N–O stretching and N–H bending vibrational modes from amide I and amide II, respectively.^11,12,26^

**Figure 1:**
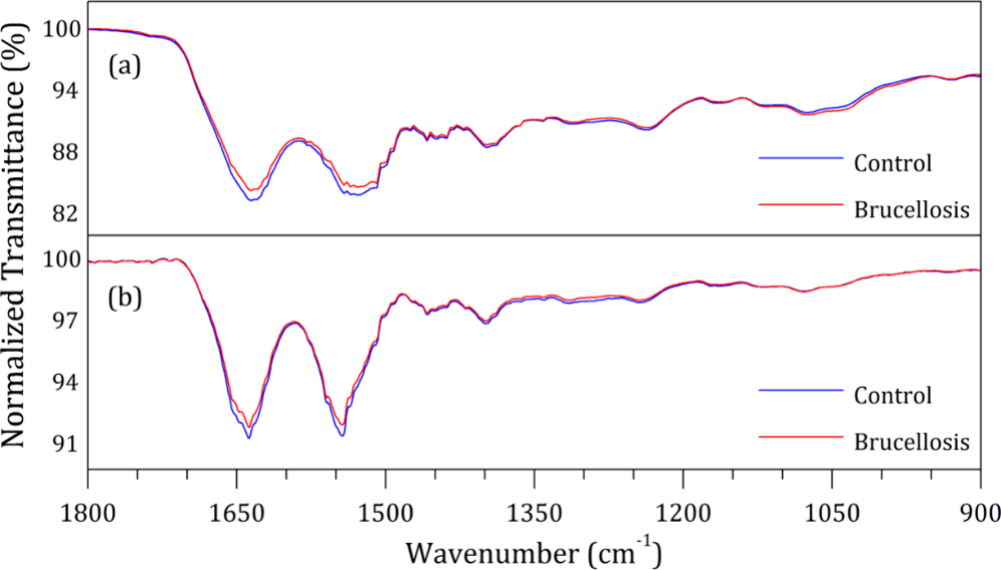
Superimposed FTIR-SNV spectra for the bovine blood serum control group (blue-colored trace) and the B. abortus-infected group (red-colored trace)—called brucellosis. (a) Dried sample (upper spectral set) and (b) liquid sample (lower spectral set). For each kind of sample, the control and brucellosis group spectra were superimposed for direct comparison.

Both sample groups exhibit the same vibrational bands with closely matching positions and relative intensities. The spectra of the dried samples are approximately 6 times more intense than those of the liquid samples, primarily due to concentration differences. Notably, the liquid samples display a distinctive pattern: there are two strong bands in the 1650–1500 cm^–1^ range, while the weaker bands in the 1200–1000 cm^–1^ range are almost imperceptible.

Since dried samples yield a more intense signal, we anticipated that this data set could offer more precise characteristics for sample characterization and differentiation between groups. In contrast, the low-intensity spectra of the liquid samples may complicate the differentiation process as subtle variations in the spectra could be attributed to inherent data quality issues rather than actual compositional differences.

More importantly, no significant difference between the spectral groups for each kind of sample can be observed. The same bands with similar characteristics are present for both groups. The main prominent band identified for both groups was assigned for amides I and II from proteins, around 1638 and 1543 cm^–1^, respectively, followed by minor vibrational bands at 1458 cm^–1^ (CH_2_ symmetric bending) from lipids; 1398 cm^–1^ (COO^–^ symmetric stretching) and 1317 cm^–1^ (amide III) from proteins; 1243 cm^–1^ (PO_2_^–^ asymmetric stretching) from the phosphate band; 1168 cm^–1^ (C–OH asymmetric stretching) from proteins; 1081 cm^–1^ (PO_2_^–^ symmetric stretching) from the phosphate band; and 930 cm^–1^ (C–C stretching) from carbohydrates.^11,12,27^

The FTIR-SNV spectra for dried samples and the FTIR-SNV-IFFT spectra for liquid samples were analyzed by principal component analysis (PCA). Figure 2 summarizes the main results found for dried (Figure 2a,b) and liquid (Figure 2c,d) samples. The normalized score plot for the FTIR-SNV spectra of bovine dried blood serum (Figure 2a) demonstrates a clear tendency for separation using only two principal components (PCs), which account for 92.3% of the data variance. Both the control group (blue circles) and the brucellosis group (red circles) exhibit high dispersion along both PC1 and PC2, reflecting the significant intragroup variance due to the inherent variability of the animals studied. Despite this, the variance between the control and brucellosis groups reveals a distinct separation trend along the opposite diagonal. This separation trend indicates a promising condition for building a predictive model using SVM algorithms.

**Figure 2:**
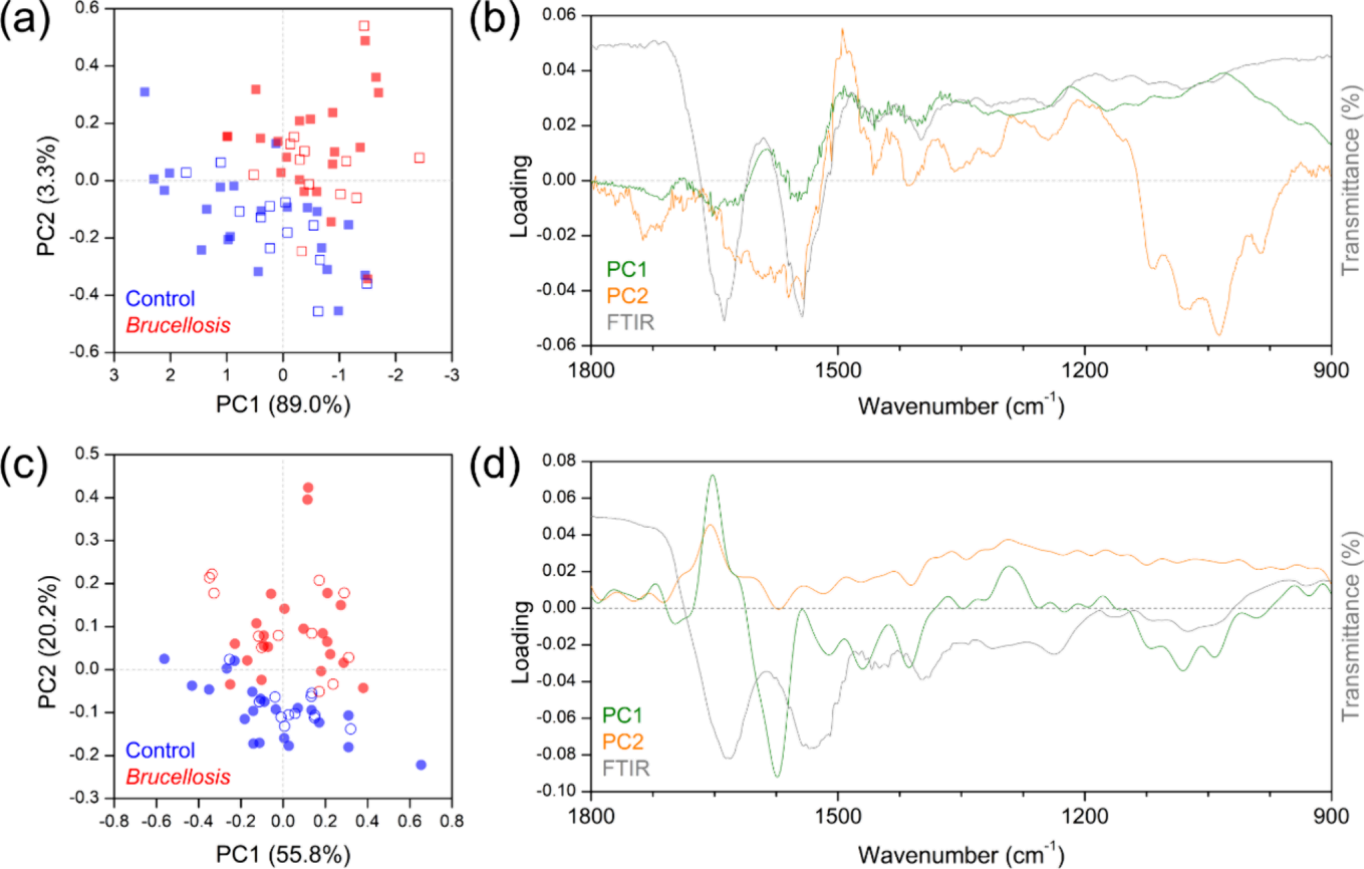
Principal component analysis results from (a, b) FTIR-SNV dried sample spectra and (c, d) FTIR-SNV-IFFT liquid sample spectra. B. abortus-infected group (red circles) and control (noninfected) group (blue circles). Fully colored symbols represent the samples included in the leave-one-out cross-validation (LOOCV) process, while hollow symbols represent independent validation samples, which were not used during model training but were later classified by the trained model to evaluate its generalization performance. The score plot on the left side and loading plot on the right side.

By analyzing the loading plot in Figure 2b, we can correlate the data variance with the original data, supporting the chemical significance of the analysis. The PC1 loading shows a significant contribution from the amide I and II bands between 1650 and 1500 cm^–1^, followed by contributions around 1400, 1200, and 1050 cm^–1^. Additionally, the PC2 loading also highlights a major contribution from the amide I and II bands, with further contributions between 1500 and 1300 cm^–1^ and a notable contribution in the 1150–950 cm^–1^ range.

The normalized score plot for the FTIR-SNV-IFFT spectra of bovine liquid blood serum (Figure 2c) also demonstrates a clear tendency for separation using only two principal components (PCs), which account for 76% of the data variance. The expected high dispersion along both PC1 and PC2 for both the control group (blue circles) and the brucellosis group (red circles) was also observed. Despite this, the variance between the control and brucellosis groups reveals a distinct separation trend along the diagonal. In the same way, this separation trend suggests a promising possibility for building a predictive model using SVM algorithms.

By analyzing the loading plot in Figure 2d, we can observe that the same data variance present in the dried sample is evident, although the intensity has changed considerably. Significant differences are noted in the 1500–1700 cm^–1^ range, where prominent protein bands, including amides I and II, are notable. All contributions to data variance are strongly correlated with the FTIR spectra and changes in the molecular composition of the blood serum. These changes are due to the immune response involving new molecules associated with macrophages, neutrophils, lymphocytes, antibodies, antigens, and cytokine cells.^4–9^

The separation frontier observed in Figure 2a,c cannot be approximated by a purely linear function, as evidenced by the intermixing of red and blue points within each other’s regions. This suggests the need to evaluate quadratic and cubic functions in addition to adjusting hyperparameters. Adjusting hyperparameter *C* in an SVM controls the trade-off between margin width and classification error, thereby influencing the complexity and smoothness of the decision hyperplane. However, when the score plot shows this clear tendency of group separation, very promising results are expected for machine learning algorithms to build successful prediction models.^26,27^

Figure 3 shows the accuracy values obtained in the LOOCV and validation tests using the SVM algorithm with different functions and hyperparameter (*C*) values. No combination of the SVM function, hyperparameter, or sample nature achieved 100% accuracy. For all tests, we limited the number of principal components (PCs) to two, consistent with the score plot shown in Figure 2. Overall, both dried and liquid samples provided a method for brucellosis diagnosis with an accuracy above 90%. Although the highest accuracy for dried samples was observed with the cubic SVM, the quadratic SVM was selected as the optimal model due to its balanced performance across training and validation. Similarly, for liquid samples, the linear SVM with *C* = 100 demonstrated a strong classification performance with stable results between LOOCV and validation.

**Figure 3:**
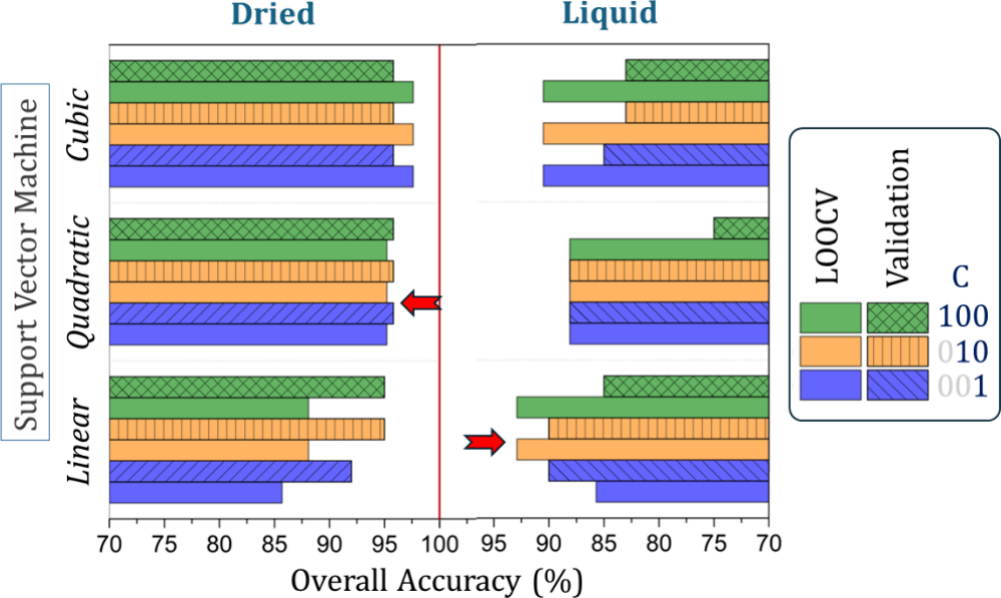
Overall accuracy results obtained in the LOOCV (full color) and validation (line pattern) tests for the support vector machine (SVM) algorithms. Only the first two PCs were explored according to the function (linear, quadratic, and cubic) and the hyperparameter *C* (1, 10, and 100) in the 1800–900 cm^–1^ range for dried and liquid blood serum samples. The red arrows indicate the models that achieved both high accuracy and minimal difference between LOOCV and validation tests, ensuring greater robustness and generalizability.

We have an unbalanced group of samples (60/40), which may result in slight deviations in the overall accuracy between LOOCV and validation tests. However, our goal is to develop a more stable prediction model, which can be achieved by using a small number of PCs with high chemical significance. This approach offers simpler mathematical interpretation and, importantly, similar overall accuracy in both LOOCV and validation tests; here, we consider the best prediction models to be those that exhibit the smallest accuracy difference between leave-one-out cross-validation (LOOCV) and the validation test. Additionally, the accuracy from the validation test must not exceed that obtained from LOOCV. For such conditions, the best prediction model for dried samples was found by using the quadratic SVM with any hyperparameter *C*, which exhibits an overall accuracy of around 95% for LOOCV and validation tests (Figure 4a). The linear SVM with hyperparameter *C* = 100 for liquid samples was shown to be the best choice, with an overall accuracy of around 92% for LOOCV and validation tests (Figure 4c).

**Figure 4:**
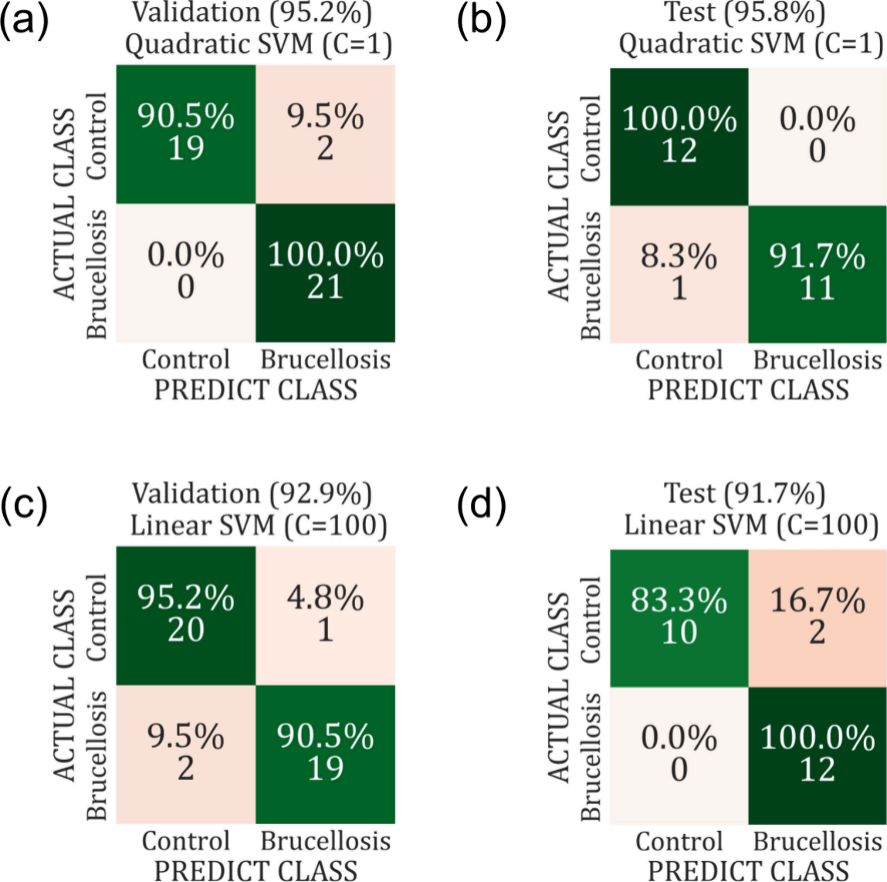
Confusion matrices showing the classification performance of the SVM algorithm using two principal components (PCs) for dried and liquid blood serum samples. The right-side matrices represent the results from the LOOCV test (using 60% of the data), while the left-side matrices show the external validation results (using the remaining 40%). The PCA data were extracted from FTIR blood serum spectra in the 1800–900 cm^–1^ range. In the figure, panels (a, b) correspond to dried serum samples, whereas panels (c, d) correspond to liquid serum samples.

The PCA analysis results clearly suggest a significant difference between the two groups analyzed. This leads to an easy interpretation of the differences caused by the immune response to the B. abortus infection. Additionally, it facilitates the construction of a predictive model using machine learning that is simpler and more intuitive, such as models based on the SVM algorithm, without requiring a more robust data analysis for this task. The correlation with the original data observed from the PCA analysis further reinforces the results obtained in the supervised validation tests presented in the right column of Figure 4. The control group used in this study consists of animals tested for brucellosis exclusion but monitored for overall animal health and not necessarily free from other diseases. The robustness of the proposed method now requires analysis with other disease groups that have similar immune response mechanisms to verify if this data analysis routine can be safely implemented as a routine test.

The current diagnostic tests take up to 1 week, leading to empiric antibiotic prescriptions. To avoid future problems, rapid genomic tests are being developed to identify pathogens and resistance genes within a day to guide more targeted antibiotic use and improve antimicrobial stewardship.^28^ Here, we present a potential contribution to implementing photodiagnosis tests to help in this important task of disease control and surveillance.

Bovine brucellosis is an inherently chronic disease, characterized by a slow progression of clinical signs following infection that may take months or even years to manifest. In some instances, such as in infected male cattle, clinical signs might not appear at all. The primary challenge in diagnosing bovine brucellosis lies in identifying asymptomatic animals to facilitate their removal from herds, thereby reducing disease transmission and enabling herd sanitation over time, ultimately achieving a “disease-free” status.^29^

In this study, samples were collected from asymptomatic animals. According to current Brazilian legislation, females aged 24 months and older, vaccinated up to 8 months of age, must undergo diagnostic testing for bovine brucellosis, regardless of symptomatology.^17^ Consequently, routine herd management in Brazil mandates that all females meeting this age requirement be subjected to serological tests for the disease. Animals identified as reactive must then be culled or subjected to sanitary slaughter. Thus, this study aimed to evaluate the proposed diagnostic method under the same conditions as those employed in the routine bovine brucellosis diagnosis.

Moreover, as in any infectious disease, the stage of infection is directly correlated to the serum antibody titer. In this study, all samples underwent traditional serological tests (BAAT, 2-ME, and SAT), which are established and recommended for bovine brucellosis diagnosis.^17^ In samples classified as positive, antibodies were consistently detected using conventional methods, indicating an adequate humoral immune response in infected animals.

For samples classified as negative, it is plausible that some originated from animals in the very early stages of infection (less than approximately 15 days), during which a detectable humoral immune response has not yet developed. In such cases, further studies involving experimental infections and longitudinal animal monitoring could provide insights into the proposed diagnostic method’s capacity for early detection. However, given the pathogen’s nature and associated biological risks, such studies can only be conducted in biosafety level 3 (BSL-3),^30^ facilities equipped for large animal experimentation unavailable in Brazil. Alternatively, experiments could be conducted using mice in appropriately equipped facilities that are more widely available. Nonetheless, the humoral immune response in mice does not necessarily reflect that of bovines, and any conclusions drawn under such conditions would be speculative at best.

## Conclusions

The combination of FTIR spectroscopy and machine learning enables the accurate differentiation of serum samples from bovine brucellosis-infected and noninfected animals. In dried serum samples, the method achieved 95.8% accuracy, 91.7% sensitivity, and 100% specificity, while for liquid serum samples, it reached 91.7% accuracy, 100% sensitivity, and 83.3% specificity. Notably, both approaches outperformed traditional gold-standard diagnostic tests. Furthermore, the ability to analyze liquid serum without the need for an additional drying step enhances the method’s practicality, allowing for rapid and efficient health monitoring of cattle. This advancement facilitates better disease control and contributes to the prevention of bovine brucellosis transmission to humans.

The FTIR-based method developed in this study offers a rapid, cost-effective, and highly accurate diagnostic approach, making it particularly valuable as a screening tool for the early detection of asymptomatic cases. Providing a preliminary assessment in just a few minutes can aid in decision-making and optimizing disease control strategies. However, as with any screening test, confirmatory laboratory testing using gold-standard methods (such as serological or molecular assays) remains necessary for a definitive diagnosis. Integrating this FTIR-based approach into routine surveillance programs could significantly improve diagnostic efficiency by reducing the number of samples requiring confirmatory testing while ensuring reliable detection of brucellosis.

## Data Availability

Data will be shared upon request.

## Notes

All participants were informed and consent for publication was obtained.
